# Anatomical Outcome of Vitreoretinal Surgery Using Temporary Keratoprosthesis and Replacement of the Trephined Corneal Button for Severe Open Globe Injuries: One-Year Result

**DOI:** 10.1155/2014/794039

**Published:** 2014-07-01

**Authors:** Hui-Jin Chen, Chang-Guan Wang, Hong-Liang Dou, Xue-Feng Feng, Kang Feng, Yun-Tao Hu, Yi-Min Xu, Zhi-Zhong Ma

**Affiliations:** Department of Ophthalmology, Key Laboratory of Vision Loss and Restoration, Ministry of Education, Peking University Third Hospital, 49 HuaYuan BeiLu, Haidian District, Beijing 100191, China

## Abstract

In this case series of 74 patients with coexisting vitreoretinal injury and severe corneal opacification, after temporary keratoprosthesis (TKP) assisted pars plana vitrectomy (PPV), an allograft corneal transplant was not performed at the same time; instead, the patient's trephined corneal button was sutured back. One year after the surgery, if intraocular pressure of the injured eyes was above 8 mmHg, removing silicone oil was attempted, and penetrating keratoplasty could be performed. Finally, 10 eyes (13.5%) were enucleated due to atrophia bulbi; 46 eyes (62.2%) were silicone-oil sustained; 15 eyes (20.3%) were anatomically restored; and 3 eyes (4.0%) experienced recurrent retinal detachment. These figures only demonstrate a small percentage of the injured eyes in our series, which have PKP indications. It is a practical option to suture back the patient's trephined cornea following a TKP assisted PPV; keratoplasty was reserved for selected cases.

## 1. Introduction

A severe ocular injury sometimes results in an anterior segment anomaly and a posterior segment disturbance, simultaneously. A variety of corneal injuries can cause an opaque cornea that impedes the visualization of the fundus during vitreoretinal surgery. For this circumstance, an endoscopy-assisted vitrectomy is one solution [1], and the application of temporary keratoprosthesis (TKP) is another solution [2–5]. A penetrating keratoplasty (PKP) procedure is usually performed after a pars plana vitrectomy (PPV) using TKP. However, the long-term outcomes of this triple procedure (PPV + TKP + PKP) were not so optimistic for the traumatized eyes [2–5]. The main reasons for these unfavorable results were ciliary body malfunction and secondary graft failure [3].

In our center, a different strategy was used in treating patients with coexisting vitreoretinal injury and severe corneal opacification. After a TKP assisted PPV, an allograft corneal transplant was not performed at the same time; instead, the patient's trephined corneal button was sutured back. PKP was later considered for selected cases. This surgical method was previously not reported in the large prospective case series. Herein, we present the anatomic outcome of this procedure (TKP + PPV + replacement of the trephined cornea [RTC]).

## 2. Patients and Methods

All subjects in this study were selected from the database of the eye injury vitrectomy study (EIVS), which was established in January 1997. The details and protocols of EIVS have been introduced in previous publications [6] and are briefly described here. EIVS is a hospital-based prospective cohort study. Six tertiary hospitals in China have successively participated in it. The type and zone of the injury conform to the recommendations of the United States Eye Injury Registry and the International Society of Ocular Trauma (Birmingham Eye Trauma Terminology (BETT [7])); “A System for Classifying Mechanical Injuries of the Eye” [8]. The intraocular area of the injured eye was examined and assessed during vitreoretinal surgery, and the results were recorded by the chief surgeon as baseline information for the “register of eye injury” form. Follow-up information was continuously added into the form later on. All of these data were entered into the EIVS electronic database using Epidata (The EpiData Association, Odense, Denmark).

For this study, we selected cases from January 2008 to June 2012 in the EIVS database. The inclusion criteria include (1) eyes that suffered from an open globe injury with retinal detachment; (2) TKP + PPV + RTC was performed; (3) the injured eye was tamponade with silicone oil; and (4) the postoperative follow-up was no less than twelve months. The exclusion criteria are patients who have missing records; those without complete follow-up information; those who suffered open globe injuries with no retinal detachment; those who had an allograft transplant during PPV due to corneal infection or severe corneal destruction; those with gas tamponade eyes; and those who had a follow-up in less than twelve months.

**Figure 1:**
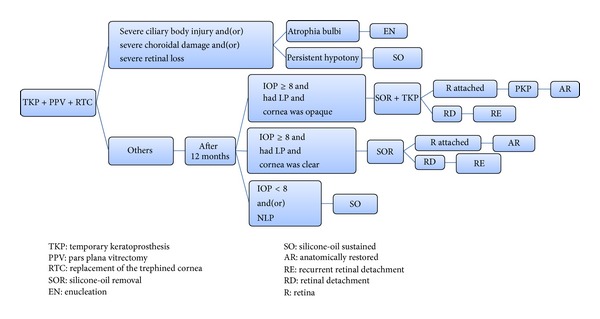


The baseline information includes the preoperative and intraoperative assessment of the injured eye. The preoperative examinations include posttraumatic vision, intraocular pressure (IOP), and B-scan ultrasonography. Most of the relevant intraoperative assessments include type and zone of the injury, causes of corneal opacification, the length of the wound, extension of the severe ciliary body injury (defined as inability to recognize the structure of ciliary processes in at least two quadrants), severe choroidal damage (defined as a communication between the vitreous cavity and the suprachoroidal cavity caused by choroidal rupture, and which is surgically unrepairable), severe loss of the retinal tissue (defined as retinal loss in at least two quadrants), and the retinal reattachment rate during an operation. The follow-up information includes a postoperative vision, IOP, and corneal transparency and the anatomical outcome of the injured eyes, which were categorized into four types according to the following flowchart (Figure 1): anatomically restored (defined as IOP no less than 8 mmHg, retina is reattached, and silicone oil is removed); silicone-oil sustained (defined as IOP less than 8 mmHg, silicone oil cannot be removed, and the eyes are permanently tamponade with silicone oil); enucleated due to phthisis bulbi; and recurrent with retinal detachment.

### 2.1. Surgical Procedures

Under general anesthesia, the eyes were prepared for a standard three-port vitrectomy. The pathologic cornea was trephined and the corneal button was protected by a viscoelastic material and kept in a humid container. Ocular Landers wide field temporary keratoprosthesis (Ocular Instruments, USA) was sutured with a 6-0 vicryl suture (Johnson & Johnson Services, Inc., USA). In addition to vitrectomy, surgical procedures may include synechiolysis of the anterior segment, cyclopexy, iridoplasty, lensectomy, membrane peeling, retinotomy and retinectomy, foreign body extraction, subretinal hemorrhage removal, use of perfluorocarbon liquids, and endophotocoagulation. At the end of the surgery, instead of an allograft transplant, the patient's trephined corneal button was sutured back. All eyes were tamponade with silicone oil.

## 3. Results

74 eyes of 73 patients met the criteria in this study (66 male and 7 female). The mean age of the patients was 34.6 ± 15.8 years old (range, 5~69). For posttraumatic vision, there was no light perception (NLP) in 54 eyes; light perception (LP) in 17 eyes; and hand motion (HM) in 3 eyes. The mean posttraumatic IOP was 4.7 ± 2.7 mmHg (range, 2~14). The average interval of injury and vitrectomy (IOIV) was 23.8 ± 12.4 days (range, 5~60). 

*Intraoperative Assessment of the Injured Eye*. Types of eye injury are summarized in Table 1. All eyes suffered from zone III injuries. Causes of corneal opacification are summarized in Table 2. The mean length of the wound was 12.9 ± 6.9 mm (range, 5~30). 45 eyes (60.8%) had severe ciliary body injury. 33 eyes (44.6%) had severe choroidal damage. 25 eyes (33.8%) had severe retinal tissue loss. 5 eyes (6.8%) had total retinal loss. The retina of 2 eyes (2.9%) could not be reattached during the surgery. 

**Figure 2:**
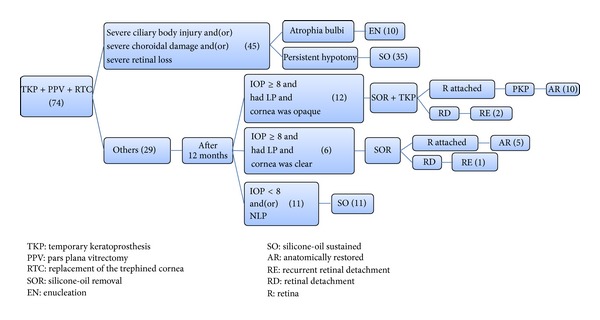


*Patient's Follow-Up*. 10 eyes (13.5%) were enucleated due to atrophia bulbi during follow-up. One year after the TKP + PPV + RTC procedure, the vision results were NLP in 28 eyes (43.8%); LP in 18 eyes (28.1%); HM in 13 eyes (20.3%); and finger count in 5 eyes (7.8%). 16 out of 54 eyes (29.6%) improved from preoperative NLP to postoperative LP; 29 eyes (45.3%) had improved vision after the TKP + PPV + RTC procedure; and 35 eyes (54.7%) remained unchanged. The mean postoperative IOP during the final visit was 8 ± 3.09 mmHg (range, 5~16). The anatomical outcome of the injured eyes is shown in Figure 2. 10 eyes (13.5%) were enucleated due to atrophia bulbi; 46 eyes (62.2%) were silicone-oil sustained; 15 eyes (20.3%) were anatomically restored; and 3 eyes (4.0%) experienced recurrent RD.

## 4. Discussion

When treating patients with coexisting corneal opacification and vitreoretinal disorders, the triple procedure (PPV + TKP + PKP) has been commonly used [2–5]. However, open globe eye trauma is a unique clinical entity since the injured eye would usually have a complex and multiple intraocular structure disorganization. Special attention must be paid to this situation; and the routine triple procedure (PPV + TKP + PKP) may need some modifications for the injured eyes. For instance, in our practice, an allograft was not transplanted during an exploratory vitrectomy at the acute phase of the injury. Instead, the patient's opaque cornea was sutured back. Several reasons justified this strategy.

First of all, for severely injured eyes, postoperative persistent hypotony is common; and a longstanding or permanent silicone-oil tamponade may be needed. If PKP is performed on such eyes, the success rate will dramatically be reduced due to insufficient nourishment of the cornea, which is caused by an inadequate aqueous flow and an unavoidable silicone oil-endothelium contact [9–11]. Roters et al. [3] reported a series of 34 severely injured eyes that underwent the PPV + TKP + PKP procedure; 8 eyes were considered phthisical and 10 eyes had a longstanding hypotony. During the follow-up period, graft opacification with corneal decompensation was found in 29 of 34 eyes. The author pointed out that there is a higher risk of graft failure if silicone oil is not removed. In their case series, 21 eyes (62%) had silicone oil-corneal endothelium contact; and all of those grafts failed. In our case series, 46 eyes (62.2%) experienced persistent hypotony and the silicone oil could not be removed; and 10 eyes (13.5%) were enucleated due to atrophia bulbi. Only 15 eyes (20.3%) were anatomically restored. These figures only demonstrate a small percentage of the injured eyes in our series, which have PKP indications.

Secondly, hematocornea, which is a common reason for corneal opacification after a severe ocular trauma, is sometimes reversible. Brodrick [12] observed that 6 eyes had corneal blood staining due to contusion injuries, which occurs in traumatic hyphema cases. He observed that the cornea became completely clear in 4 out of 6 eyes, although the processes may take a long time. In our case series, hematocornea accounts for 67.6% of corneal opacification. During the one-year follow-up, all eyes with hematocornea showed some degree of clearing; and in 6 eyes, the cornea became clear enough; hence, PKP was no longer necessary. For these cases, if allografts were hastily performed, the patients would have suffered a series of unnecessary troubles, such as long-term use of topical steroids and immunosuppressive agents, and a lifetime vigilance for graft rejection. Therefore, eyes with corneal blood staining will be observed for at least one year to see if it can resolve by itself.

Lastly, the injured eyes are usually still congested and inflamed at the time of vitrectomy, which increases the risk of graft failure. Roters et al. [3] found that a transplant failure was more frequent for eyes that were grafted within 8 weeks of trauma. Besides, in areas with donor tissue scarcity, allograft is often not available for the limited time frame of vitrectomy surgery. Suturing back the patient's trephined corneal button allows the PKP to be performed in a relatively quiet eye and provides sufficient time for waiting for the donor.

There is also a limitation in our surgical strategy. It is undeniable that suturing back the patient's opaque cornea may cause some inconvenience in observing the fundus after the surgery. However, in many patients, the transparency of the cornea gets better over a period of time after surgery, and the fundus can usually be examined through the area of the transparent cornea. For patients with a completely opaque cornea, there are two major issues we can take into account when determining the visual potential of the eye or making plans for additional surgery: one is the previous surgical record with detailed description of the status of the retina and the other is the level of IOP and the sensitivity of light perception and light projection postoperatively. B-scan and endoscopy can be used to help determine the status of the retina.

In conclusion, from this large case series with a coexisting corneal and vitreoretinal injury, we found that only 20.3% of the eyes can be anatomically restored and have real value for PKP. There was also a high incidence (62.2%) of persistent hypotony and longstanding silicone-oil tamponade for severely injured eyes. Corneal blood staining was the most common reason (67.6%) for using TKP. During follow-up, all eyes with hematocornea showed some degree of clearing. Therefore, instead of using the triple procedure (PPV + TKP + PKP) as a routine treatment for every patient with coexisting vitreoretinal injury and severe corneal opacification, we advocate suturing back the patient's trephined cornea during the primary procedure and perform PKP on a later stage, as a secondary procedure for carefully selected cases.

## Figures and Tables

**Table 1:** Types of eye injury.

	Eyes	%
Rupture	55	74.3
Penetration	7	9.4
Perforation	2	2.7
IOFB	5	6.8
Open-globe mixture	5	6.8

**Table 2:** Causes of corneal opacification.

	Eyes	%
Corneal blood staining	50	67.6
Large edematous corneal wounds	20	27
Corneal explosive injury with multiple intracorneal foreign bodies	4	5.4
